# Author Correction: Heterogeneous rarity patterns drive price dynamics in NFT collections

**DOI:** 10.1038/s41598-022-20027-8

**Published:** 2022-09-12

**Authors:** Amin Mekacher, Alberto Bracci, Matthieu Nadini, Mauro Martino, Laura Alessandretti, Luca Maria Aiello, Andrea Baronchelli

**Affiliations:** 1 Department of Mathematics, University of London, London, EC1V 0HB UK; 2 Elliptic Inc., London, UK; 3 IBM Research, Cambridge, MA USA; 4 Technical University of Denmark, 2800 Kgs. Lyngby, Denmark; 5 IT University of Copenhagen, Copenhagen, Denmark; 6 UCL Centre for Blockchain Technologies, University College London, London, WC1E 6BT UK; 7 The Alan Turing Institute, London, NW1 2DB UK

Correction to: *Scientific Reports* 10.1038/s41598-022-17922-5, published online 16 August 2022

In the original version of this Article, Amin Mekacher was incorrectly listed as the corresponding author. The correct corresponding author for this Article is Andrea Baronchelli. Correspondence and request for materials should be addressed to abaronchelli@turing.ac.uk.

The original Article has been corrected.

