# Supplementary material for: Heterogeneous rarity patterns drive price dynamics in NFT collections
Source: Sci Rep. 2022 Aug 16;12:13890. doi: 10.1038/s41598-022-17922-5 (PMC9381539; doi:10.1038/s41598-022-17922-5)
Supplement: Supplementary file 1 — Supplementary Information. [file 41598_2022_17922_MOESM1_ESM.pdf]

Supplementary Information for ”Heterogeneous rarity patterns drive price dynamics in NFT collections”

Amin Mekacher, Alberto Bracci, Matthieu Nadini, Mauro Martino, Laura Alessandretti, Luca Maria Aiello, Andrea Baronchelli

Contents

A OpenSea market mechanisms **2**

B Generative art mechanisms **2**

C List of collections **3**

D Rarity score distributions **4**

E Supplementary figures **4**

E.1 Rarity rank results . . . 7

E.2 Currency robustness check . . . 9

E.3 Time robustness check . . . 10

E.4 Tails robustness check . . . 12

A OpenSea market mechanisms

For a majority of NFT collectibles, the minting happens as follows. The creators offer the possibility for anyone with a wallet to generate a new NFT for a fixed price, whose attributes will be randomly selected, even though each attribute can only be given to a specific amount of NFTs. Once every NFT has been minted by the community, they are made available to their buyers, who can sell them on a marketplace afterwards.

Before releasing their collection, creators also set how much royalty they want to get from each secondary sale related to their NFTs. As such, every time a new sale happens, the royalty is deduced from the share the seller gets, as well at 2.5% of the total price that OpenSea gets from every sale taking place on their platform.

The following table details, for a few collections, the initial price at which the NFTs could be minted (gas fee, i.e., the fees required to conduct a transaction on the Ethereum blockchain, not included). Note that these transactions are not considered as sales per se by OpenSea's official API.

**Collection**	**Minting Price**
CryptoPunks	Free
Bored Ape Yacht Club	0.08 ETH
World of Women	0.07 ETH
CryptoTrunks	0.5 ETH
CryptoCorgi	First corgi to be claimed at 0.001 ETH, last one at 1.001 ETH
Sewer Rat Social Club	0.05 ETH
Rabbit College Club	0.02 ETH
Cute Pig Club	0.03 ETH
Ape Gang	Free

B Generative art mechanisms

As previously mentioned, NFT collectibles are usually generated using an algorithmic procedure, which can lead to thousands of unique tokens created with the same set of instructions [1]. However, the inner workings of the algorithms have not been shared by the creators, and can greatly differ between collections. It is therefore impossible to assess whether the rarity curves for the collections displayed in Section "Quantifying Rarity" share similarities because their algorithms follow similar steps. In the case of the CryptoPunks, members of the community have been attempting to reverse-engineer the algorithm used by Larva Labs to generate the original Punks [2], or even to replicate it [3, 4]. However, the creators never released any information on the matter, as well as any other NFT collectibles creator.

C List of collections

Collection Names				
0N1 Force	0xVampire Project	24px	8 BIT UNIVERSE	Absurd Arboretum
Adam Bomb Squad	AfroDroids By Owo	Al Cabones	AlphaBetty Doodles	AmeegosOfficialNFT
Angels of Aether	Angry Boars	AnimalWorldWar	Animathereum	Animetas
Ape Gang	Ape Harbour Yachts	ApesOfSpace	Approving Corgis	Arabian Camels
ArcadeNFT	Art Stars Club Official	Astro Frens	Astrohedz	Avarik Saga Universe
Avastars	Axolittles	BASTARD GAN PUNKS V2	BLU Blox	BULLSEUM
BYOPills	Baby Combat Bots G1	Bad Bunnies NFT	Bad Kids Alley Official	Badass Bulls
Barn Owls	Barn Owls Dino Palz	Based Fish Mafia	Bear Market Bears	Bears Deluxe
BearsOnTheBlock	Beatnik Tiki Tribe	Bit Wine	BlankFace	Blob Mob
BlockchainBikers	Bones & Bananas	Bones Club Heritage	Bonsai by ZENFT	Bored Ape Kennel Club
Bored Ape Yacht Club	Bored Mummy Baby Waking Up	Bored Mummy Waking Up	Boring Bananas Co.	Boss Beauties
BroadcastersNFT	BullsOnTheBlock	Bunker Beasts	Buzzed Bear Hideout	CHIBI DINOS
COVIDPunks!	CanineCartel	Cartlads	Catctus Collectibles	Catshit Crazy
Chads NFT	ChainFaces	Chibi Apes	Chihuahua Gang	Chill Frogs NFT
Chiptos	Chubbies	Ciphersquares Official	Citizens of Bulliever Island	Claylings
CleverGirls NFT	Cool Cats NFT	Crazy Crows Chess Club	Crazy Dragon Corps	Crazy Lizard Army
CrazySkullzNFT	Criminal Donkeys	Crumbys Bakery	CrypToadz by GREMPLIN	Cryptinieis
Crypto Cannabis Club	Crypto Corgis	Crypto Duckies	Crypto Ghosts NFT	Crypto Hobos
Crypto Hodlers NFT	Crypto Squatches	Crypto Tuners	Crypto.Chicks	CryptoFighters
CryptoFinney	CryptoMutts	CryptoPunks	CryptoSkulls	CryptoTrunks
Cunning Foxes	Cupcats Official	Cute Pig Club	CyberKongz	CyberKongz VX
CyberPunkA12	Cybergirl Fashion	Cypher City	Dapper Dinos Karma Collective	Dapper Dinos NFT
Dapper Space Collective	Dead Devil Society	DeadFellaz	DeadHeads	Deadbears Official
Deez Nuts (Official Nuts)	Degen Gang	Degenz	Delisted Tiny Punks	Derpy Birbs
Devious Demon Dudes	Dizzy Dragons	Doge Pound Puppies	DogePirates	Dogs Unchained
Dope Shibas	Dreamloops	DystoPunks	Encryptas	Epic Eagles
Ether Cards Founder	EtherGals	Ethereans Official	Etheremura	Evil Teddy Bear Club
FLUF World	FUD Monsters	FVCK_CRYSTAL//	FameLadySquas	Fang Gang
Garmers Marketverse Patrons	Fast Food Frens Collection	Fast Food Punks	Fatales	Flowtys
Floyds World	Forgotten Runes Wizards Cult	FoxyFam	Frogs In Disguise	FusionApes
Fxck Face	GLICPIXXXVER002 - GRAND COLLECTION	GOATz	GRAYCRAFT2	GRILLZ GANG
Galactic Secret Agency	GalacticApes	Galaxy Fight Club	Galaxy-Eggs	GameOfBlocks
Gator World NFT	Gauntlets	Genesis Block Art	Glue Factory Show	Goblin Goons
Good Guys NFT	Goons of Balatroon	Gorilla Nemesis	Great Ape Society	Guardians of the Metaverse
Gutter Cat Gang	Gutter Rats	HDPunks	HODL GANG	Hammys
HappyLand Gummy Bears Official	HashGuise Gen One	Hashmasks	HatchDracoNFT	Heroes of Evermore
Hewer Clan	HodlHeads	Holy Cows	HypeHippo.io	IMMORTALZ - Ambary Assassins
Incognito	Kamagang	Keplers Civil Society	KidPunks	Knights of Degen - Knights!
Koala Intelligence Agency	Koin Games Dev Squad	Kokeshi World	Krazy Koalas NFT	Lamb Duhs
Lazy Lions	Lazy Lions Bungalows	Lobby Lobsters	Lockdown Lemmings	Lonely Planet Space Observatory
Long Neckie Fellas	Long Neckie Ladies	Loopy Donuty	Loot (for Adventurers)	Lost Souls Sanctuary

Collection Names				
Lostboy NFT	Lucha Libre Knockout	Lucky Maneki	Lucky Sloths NFT	Lumps World
Lysergic Labs Shroomz	MOONDOGS ODYSSEY	Mad Banana Union	Mad Cat Militia	MaestroPups
Magic Mushroom Clubhouse	Mandelbrot Set Collection	Maneki Gang	MarsCatsVoyage	Meebits
Mighty Manateez	Mini Monkey Mafia	Minimints	MissCryptoClub	MjiBots
Monas	MonkePunks	Monkeybrix	Monster Blocks - Official	Monster Rehab 1.0
Mutant Ape Yacht Club	MutantKongz	Muttinks	My Fucking Pickle	NFT Siblings
NFTBOY: Bored Ape Racers	NOOBS NFT	Naughty Tigers Costume Club	Neon Junkies	Nice Drips
Nifty League DEGENs	Niftyriots	Non-Fungible Heroes	Notorious Frogs	ORCZ!
OctoHedz	Oddball Club (Official)	Official DogeX	Omnimorphs	OnChainMonkey
Osiris Cosmic Kids	PEACEFUL GROUPIES	PORK1984	POW NFT	PPPandas
Paladin Pandas	Panda Dynasty	Panda Golf Squad	Party Penguins	Penguin Fight Club
PinapplesDayOut	Pirate Treasure Booty Club	PixaWizards	Platy Punks - Official	PogPunks NFT
Polar Pals Bobsledding	Posh Pandas	Potato Power Club	Primate Social Society	Procedural Space
Pudgy Penguins	PunkBabies	PunkCats	PunkScapes	Purrnelopes Country Club
PyMons	Qubits On The Ice	RUUMZ	Rabbit College Club	Raccoon Mafia
Raccoons Club	RagingRhinos	Re-Genz	Ready Player Cat NFT	Reb3l Bots
Reckless Whales	RichKidsOfficial	Rickstro Frens	Rivermen	Roaring Leaders
Robotos Official	Rogue Society Bots	Royal Ceramic Club	Royal Society Chips	Royal Society of Players
Rumble Kong League	SLOTHz	STRAWBERRY.WTF	SVINS	Sad Frogs District
Sad Girls Bar	SamuraiDoge	Sappy Seals	Satoshibles	Savage Droids
Save the Martians	ScoopDog Squad	Secret Society of Whales	Sewer Rat Social Club	Shabu Town Shibas
Shaggy Sheep	Shiba Society	Sidus NFT Heroes	SingularityHeroes	Sipherian Surge
Skvullpvnks Hideout	Slacker Duck Pond	Sleeper Hits Collection Volume 1 NFT Cribs	Slimes World	Slumdoge Billionaires
Sneaky Vampire Syndicate	Soccer Doge Club	Space Dinos Club	Space Poggers	SpacePunksClub
SpaceShibas	Spookies NFT	SportsIcon Lion Club	Spunks	Standametti
Stoned Apez Saturn Club	Stoner Cats	Stranger EggZ	StripperVille NFTs	SupDucks
Super Yeti	Superfuzz The Bad Batch	Superfuzz The Good Guys	Sushiverse	SympathyForTheDevils
THE PLUTO ALLIANCE	THE SHRUNKENHEADZ	The Alien Boy	The BirdHouse	The CryptoDads
The CryptoSaints	The Doge Pound	The Fuckin' Trolls	The Goobers	The Graveyard Sale
The KILLAZ	The KittyButts	The League Of Sacred Devils	The Lost Glitches	The MonstroCities
The Moon Boyz	The NFTBirds	The Nanoz	The Nemesis Companions	The Ninja Hideout
The Project URS	The Sevens (Official)	The Shark Cove	The Soldiers Of The Metaverse	The Street Dawgs
The Unstable Horses Yard	The Vogu Collective	The Wanderers	The Wicked Craniums	The Wicked Stallions
The WolfGang Pups	The WonderQuest	The WynLambo	TheHeartProject	TheTigersGuild
Tie Dye Ninjas	Tokenmon	Tools of Rock	Top Dog Beach Club	TradeSquads
Trollz	Ugly Cuties Art Club (UCAC)	United Punks Union	Untamed Elephants	Unusual Whales
VeeFriends	Vegiemon	Vox Collectibles	Voxies	WE ARE THE OUTKAST
Waifusion	Wall Street Chads	Wanna Panda	Wannabes Music Club	Warriors of Aradena
We are Dorkis	WeMint Washington	Weird Whales	Wicked Ape Bone Club	Wicked Hound Bone Club
Wild Stag Treehouse	Winter Bears	Woodies Generative Characters	World of Women	Zunks
astroGems	bastard gan penguins	isotile Genesis Avatars	thedudes	uwucrew

D Rarity score distributions

As detailed in the main text, we use Akaike Information Criterium [5] and Maximum Likelihood Estimation to determine the distribution that best describes the rarity score distribution for each collection. We select the distribution among a subset of distributions implemented in the scipy.stats python package, requiring the distributions to be heterogeneous, continuous, and with at most 3 parameters (including location and scale). This results in choosing among the following distributions: *uniform*, *pareto*, *cauchy*, *lognormal*, *levy*, *exponential.*

E Supplementary figures

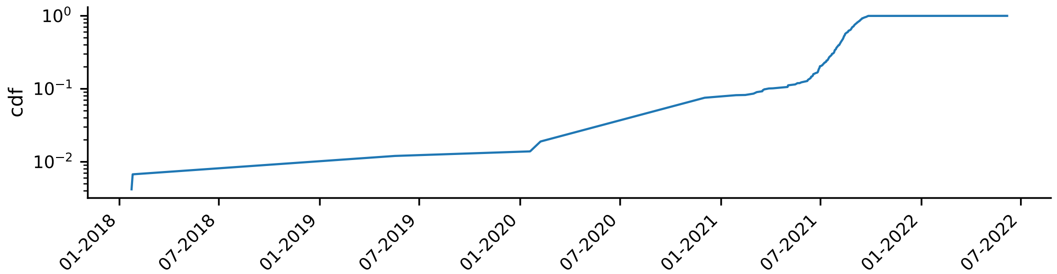

Supplementary Figure 1: **Collectible NFTs minted over time.** Distribution of the collectible NFTs considered in this analysis minted over time.

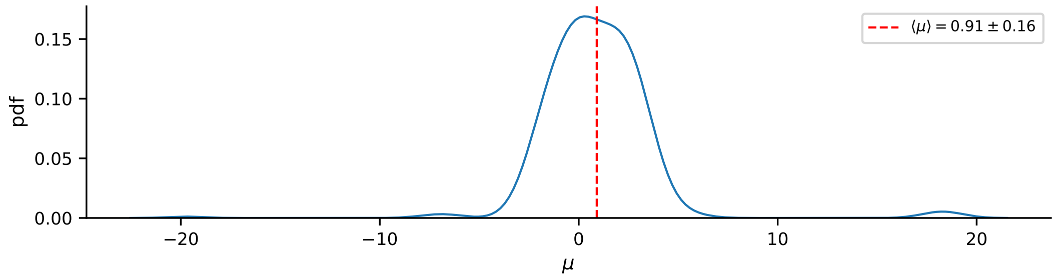

Supplementary Figure 2: **Distribution of the log-normal distribution characteristic parameter** μ**.** Distribution of the log-normal distribution parameter μ (blue line), and its average value across collections (red dashed line). The log-normal distribution $\ln(X) \sim \mathcal{N}(\mu, \sigma^2)$ captures the distribution of rarity for 90% of collections.

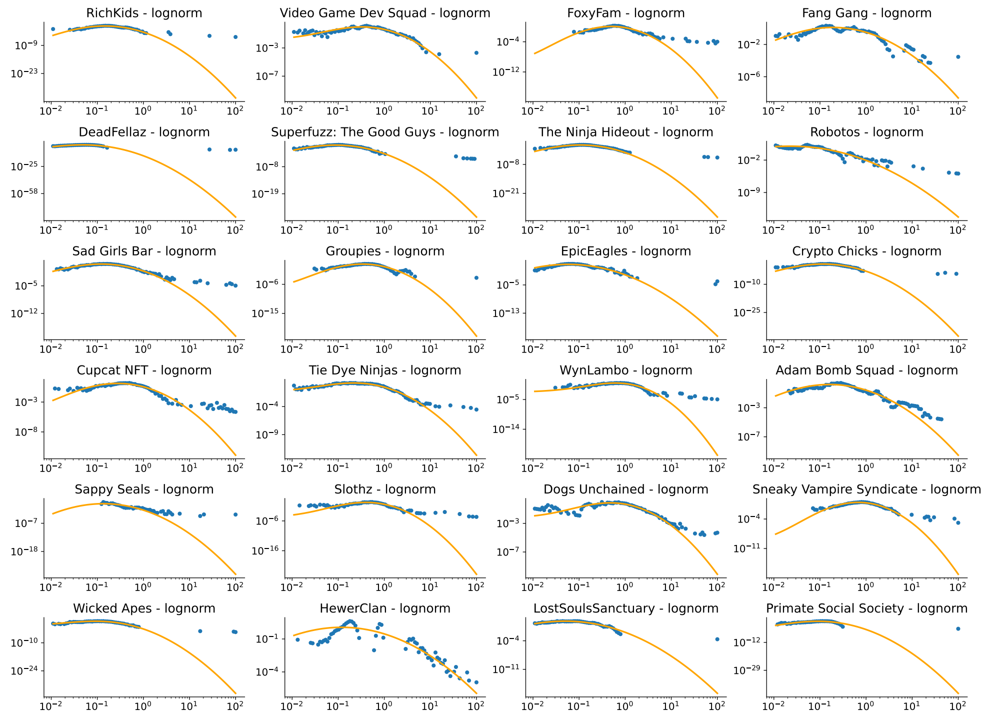

Supplementary Figure 3: **Fitting of the rarity distribution.** Distribution of the rarity score of the NFTs within several collections included in the dataset (blue dots), along with the best distribution fit computed using Maximum Likelihood Estimation and Akaike Information Criterium [5] (orange line).

E.1 Rarity rank results

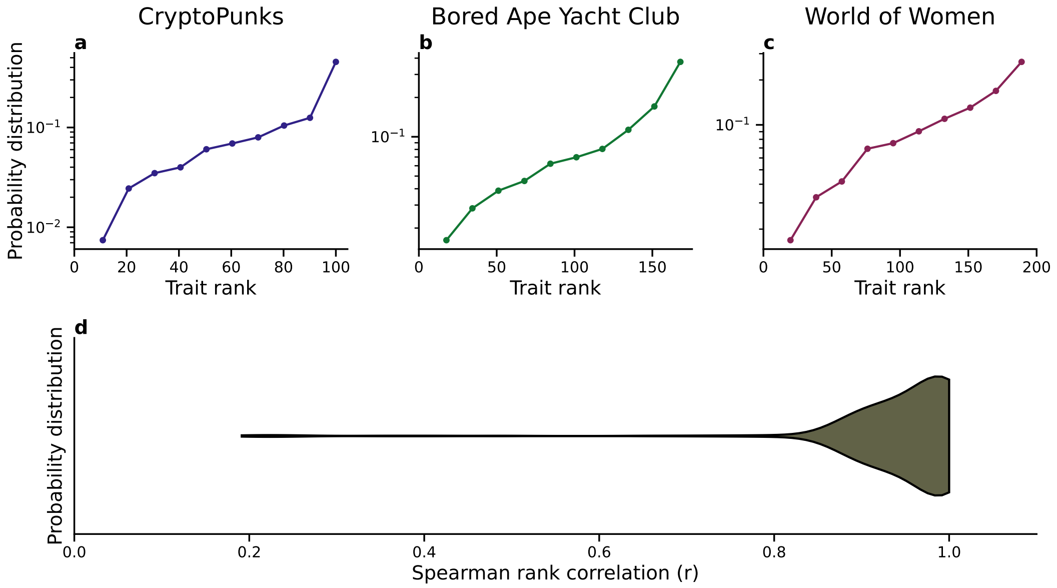

Supplementary Figure 4: **Trait Rarity Rank.** a-c) Distribution of the trait rarity rank of the NFTs within three collections: CryptoPunks (a), Bored Ape Yacht Club (b), and World of Women (c). d) Violin plot of the Spearman Rank correlation computed between the rarity rank and the number of NFTs with that rank.

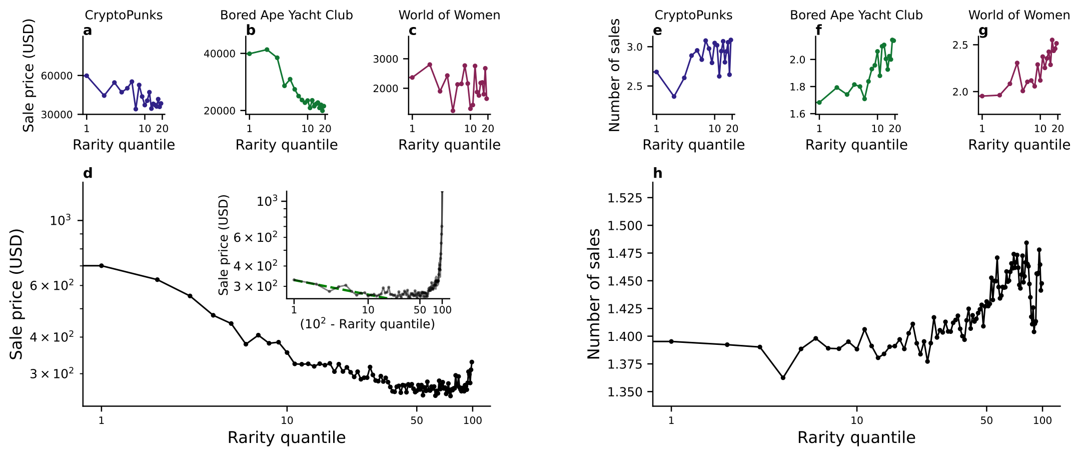

Supplementary Figure 5: **Rare NFTs have a higher financial value and circulate less on the marketplace - Analysis with the rarity rank.** Median sale price in USD (a-c) and average number of sales (e-g) by rarity quantile (with 20 quantiles considered) for three collections: CryptoPunks (a and e), Bored Ape Yacht Club (b and f), and World Women (c and g). d) Median sale price by rarity quantile (with 100 quantiles considered) considering all collections. Inset: median sale price against the quantity (100-q), where q is the rarity quantile, in log-log scale (black line) and the corresponding power law fit (green dashed line). h) Median number of sales by rarity quantile considering all collections. The NFTs are aggregated by quantile depending on their rarity rank, i.e the first quantile represents the rarest NFTs within the collection.

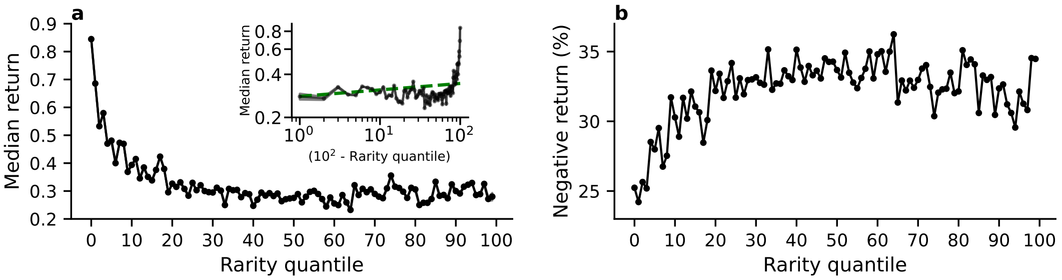

Supplementary Figure 6: **High rarity leads to higher returns, and a lower chance of a negative return - Analysis with the rarity rank.** a) Median return in USD by rarity quantile. Inset: median return against the quantity (100-q), where q is the rarity quantile in log-log scale (black line) and the corresponding power law fit (green dashed line). b) Fraction of sales with negative return in USD by rarity quantile. The NFTs are aggregated by quantile depending on their rarity rank, i.e the first quantile represents the rarest NFTs within the collection.

E.2 Currency robustness check

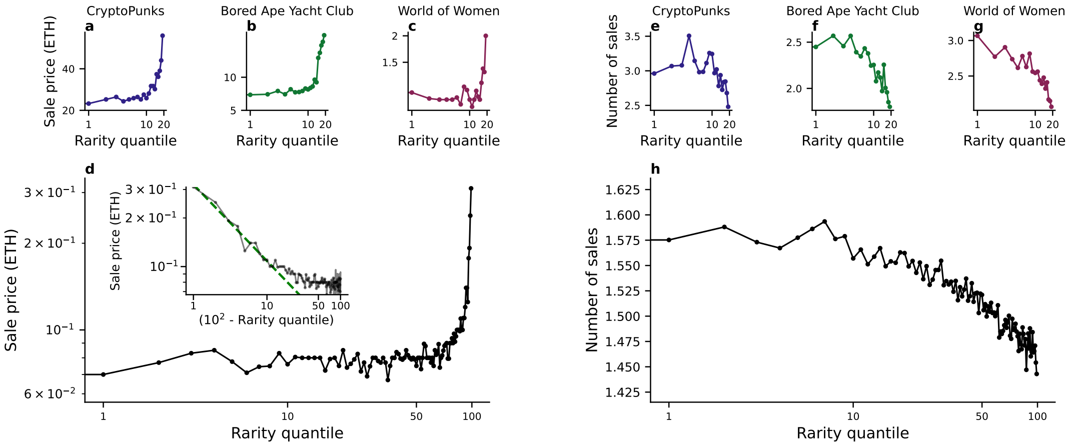

Supplementary Figure 7: **Rare NFTs have a higher financial value and circulate less on the marketplace - Price in ETH.** Median sale price in ETH (a-c) and average number of sales (e-g) by rarity quantile (with 20 quantiles considered) for three collections: CryptoPunks (a and e), Bored Ape Yacht Club (b and f), and World Women (c and g). d) Median sale price by rarity quantile (with 100 quantiles considered) considering all collections. Inset: median sale price against the quantity (100-q), where q is the rarity quantile, in log-log scale (black line) and the corresponding power law fit (green dashed line). h) Median number of sales by rarity quantile considering all collections.

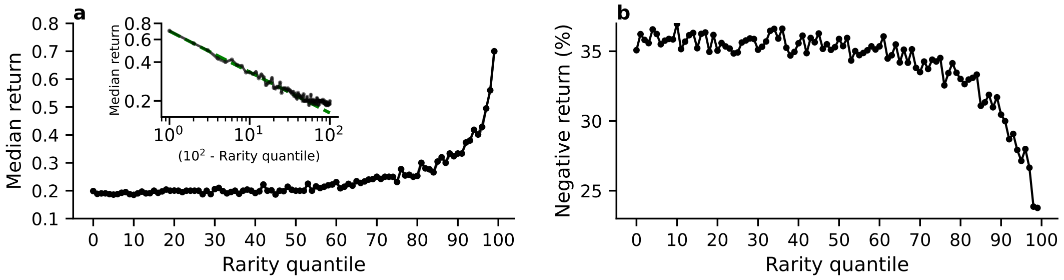

Supplementary Figure 8: **High rarity leads to higher returns, and a lower chance of a negative return - Price in ETH.** a) Median return in ETH by rarity quantile. Inset: median return against the quantity (100-q), where q is the rarity quantile in log-log scale (black line) and the corresponding power law fit (green dashed line). b) Fraction of sales with negative return in ETH by rarity quantile.

E.3 Time robustness check

To make sure that the findings we highlight in this paper are time-independent, we ran the same analysis by using only the transactions happening during specific time periods, to see whether we observe the same mechanisms within the marketplace. Therefore, we performed the analysis on the two last quarters of 2021, i.e., first on Q3 (July - September 2021) and then on Q4 (October - December 2021).

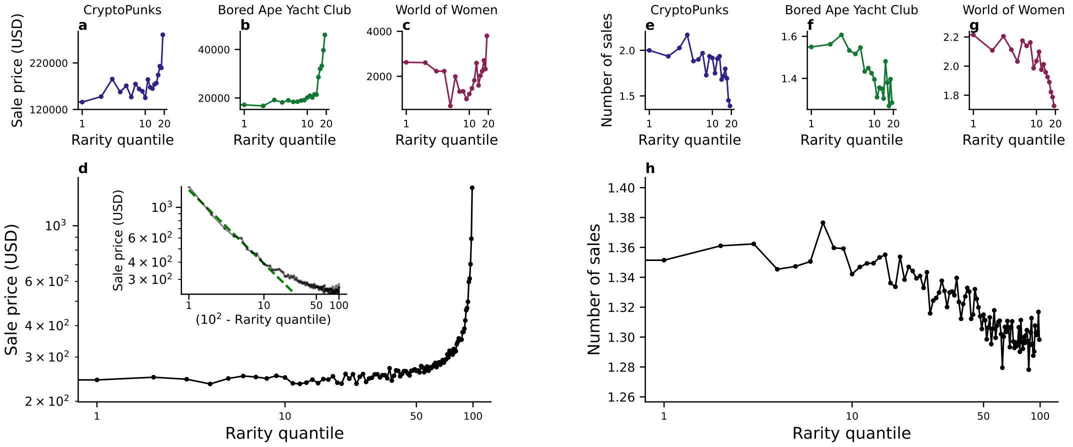

Supplementary Figure 9: **Rare NFTs have a higher financial value and circulate less on the marketplace - Analysis on Q3 2021.** Median sale price in USD (a-c) and average number of sales (e-g) by rarity quantile (with 20 quantiles considered) for three collections: CryptoPunks (a and e), Bored Ape Yacht Club (b and f), and World Women (c and g). d) Median sale price by rarity quantile (with 100 quantiles considered) considering all collections. Inset: median sale price against the quantity (100-q), where q is the rarity quantile, in log-log scale (black line) and the corresponding power law fit (green dashed line). h) Median number of sales by rarity quantile considering all collections. This analysis only takes into consideration the sales happening during Q3 2021.

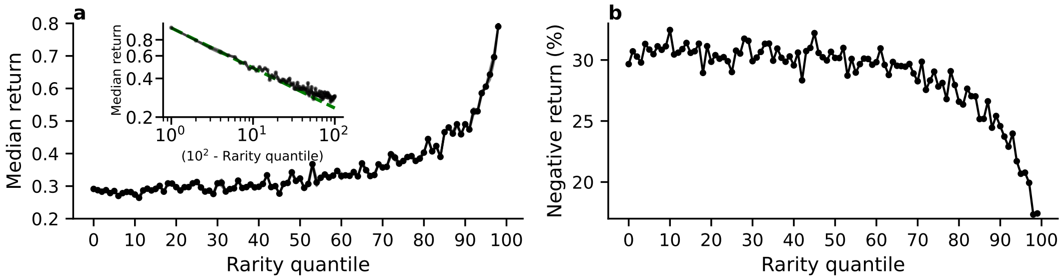

Supplementary Figure 10: **High rarity leads to higher returns, and a lower chance of a negative return - Analysis on Q3 2021.** a) Median return in USD by rarity quantile. Inset: median return against the quantity (100-q), where q is the rarity quantile in log-log scale (black line) and the corresponding power law fit (green dashed line). b) Fraction of sales with negative return in USD by rarity quantile. This analysis only takes into consideration the sales happening during Q3 2021.

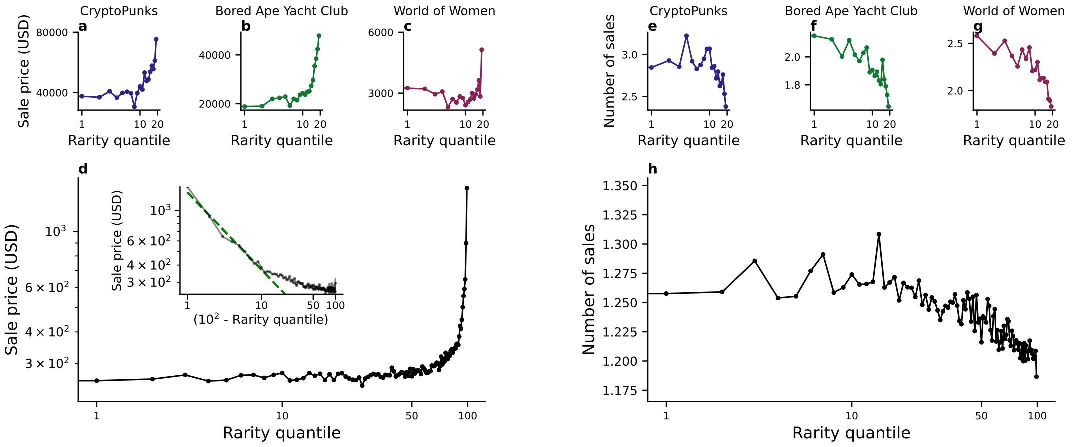

Supplementary Figure 11: **Rare NFTs have a higher financial value and circulate less on the marketplace - Analysis on Q4 2021.** Median sale price in USD (a-c) and average number of sales (e-g) by rarity quantile (with 20 quantiles considered) for three collections: CryptoPunks (a and e), Bored Ape Yacht Club (b and f), and World Women (c and g). d) Median sale price by rarity quantile (with 100 quantiles considered) considering all collections. Inset: median sale price against the quantity (100-q), where q is the rarity quantile, in log-log scale (black line) and the corresponding power law fit (green dashed line). h) Median number of sales by rarity quantile considering all collections. This analysis only takes into consideration the sales happening during Q4 2021.

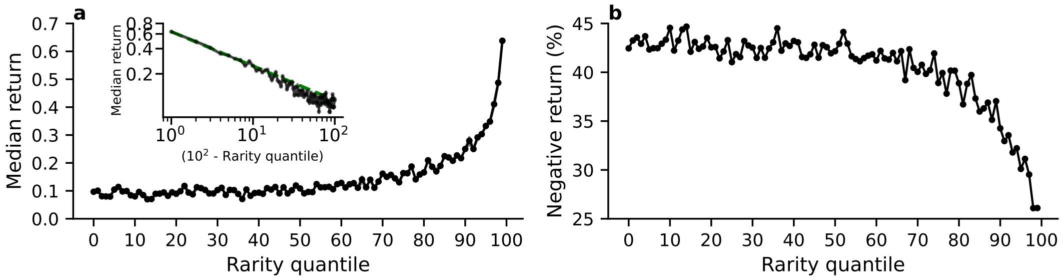

Supplementary Figure 12: **High rarity leads to higher returns, and a lower chance of a negative return - Analysis on Q4 2021.** a) Median return in USD by rarity quantile. Inset: median return against the quantity (100-q), where q is the rarity quantile in log-log scale (black line) and the corresponding power law fit (green dashed line). b) Fraction of sales with negative return in USD by rarity quantile. This analysis only takes into consideration the sales happening during Q4 2021.

E.4 Tails robustness check

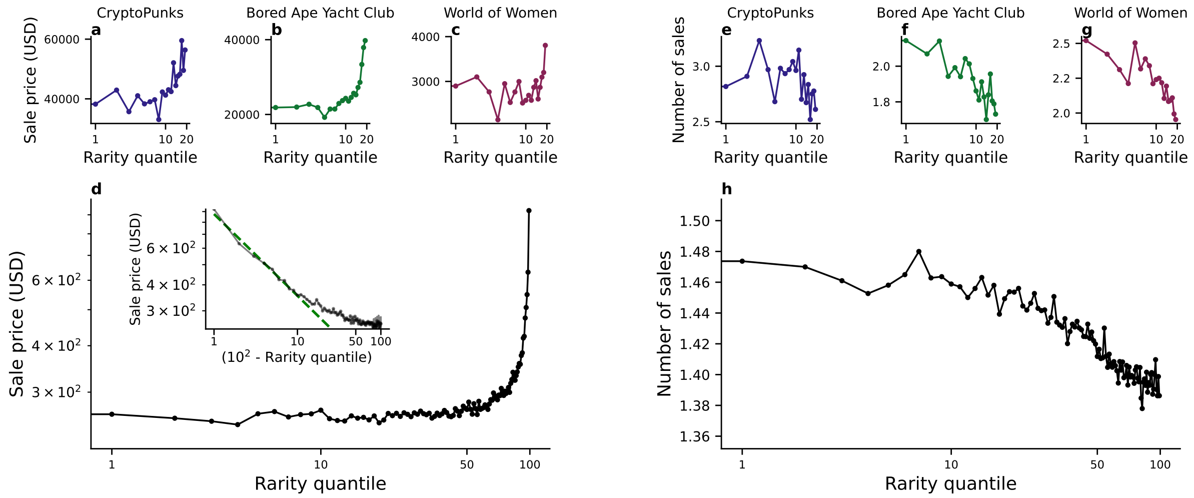

Supplementary Figure 13: **Rare NFTs have a higher financial value and circulate less on the marketplace - Analysis without the rarest and least rare NFTs.** Median sale price in USD (a-c) and average number of sales (e-g) by rarity quantile (with 20 quantiles considered) for three collections: CryptoPunks (a and e), Bored Ape Yacht Club (b and f), and World Women (c and g). d) Median sale price by rarity quantile (with 100 quantiles considered) considering all collections. Inset: median sale price against the quantity (100-q), where q is the rarity quantile, in log-log scale (black line) and the corresponding power law fit (green dashed line). h) Median number of sales by rarity quantile considering all collections. This analysis was performed after discarding the 10% rarest and least rare NFTs from each collection.

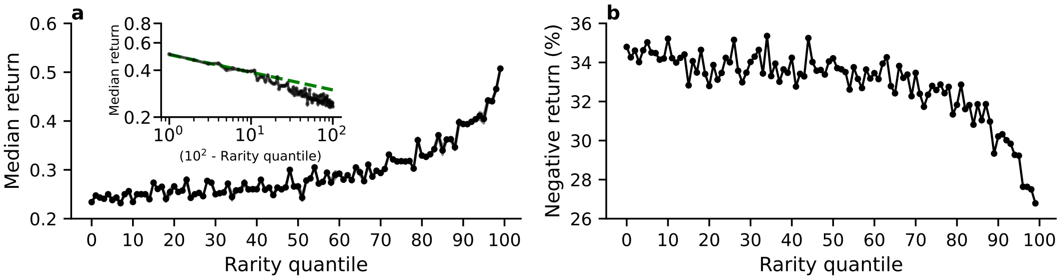

Supplementary Figure 14: **High rarity leads to higher returns, and a lower chance of a negative return - Analysis without the rarest and least rare NFTs.** a) Median return in USD by rarity quantile. Inset: median return against the quantity (100-q), where q is the rarity quantile in log-log scale (black line) and the corresponding power law fit (green dashed line). b) Fraction of sales with negative return in USD by rarity quantile. This analysis was performed after discarding the 10% rarest and least rare NFTs from each collection.

References

[1] Katharina Zeuch. An introduction to generative art nfts. https://medium.com/@datash/an-introduction-to-generative-art-nfts-35e650a0f281 Accessed March 15, 2022.

[2] The original cryptopunk. https://andrewschrbr.medium.com/the-original-cryptopunk-a852101a03ed Accessed April 4, 2022, 2021.

[3] Inside the punk art machinery - how to generate 10 000 punks (and punkettes), algorithmically - paint by numbers. https://github.com/cryptopunksnotdead/programming-cryptopunks/blob/master/03_generate.md Accessed April 4, 2022, 2021.

[4] Creating a 10k nft avatar collection: Expansionpunks step-by-step. https://medium.com/geekculture/expansionpunks-welcome-to-the-more-inclusive-punkverse-aa77e675bcf5 Accessed April 4, 2022, 2021.

[5] Eric-Jan Wagenmakers and Simon Farrell. Aic model selection using akaike weights. *Psychonomic bulletin & review*, 11(1):192–196, 2004.
